# Effects of Tai Chi combined with tDCS on cognitive function in patients with MCI: a randomized controlled trial

**DOI:** 10.3389/fpubh.2023.1199246

**Published:** 2023-08-07

**Authors:** Ying Xu, Jingfang Zhu, Hong Liu, Zhijie Qiu, Mengyuan Wu, Jiao Liu, Jingsong Wu, Jia Huang, Zhizhen Liu, Weilin Liu, Jing Tao

**Affiliations:** ^1^National-Local Joint Engineering Research Center of Rehabilitation Medicine Technology, Fujian University of Traditional Chinese Medicine, Fuzhou, Fujian, China; ^2^College of Rehabilitation Medicine, Fujian University of Traditional Chinese Medicine, Fuzhou, Fujian, China; ^3^Fujian University of Traditional Chinese Medicine, Fuzhou, Fujian, China

**Keywords:** Tai Chi, transcranial direct current stimulation, mild cognitive impairment, randomized clinical trial, cognitive function

## Abstract

**Background:**

Mild cognitive impairment (MCI) is a critical stage of dementia. Previous reviews have suggested that physical exercise combined with non-invasive brain stimulation is more beneficial for improving cognitive function. However, no targeted studies have confirmed the effect of Tai Chi combined with transcranial direct current stimulation (tDCS) on the improvement of cognitive function in patients with MCI. Thus, this randomized trial was conducted to assess the effect of Tai Chi combined with tDCS on the cognitive performance of patients with MCI.

**Methods:**

From April 2018 to February 2020, a randomized, single-blind clinical trial was conducted, involving 180 participants with MCI who were divided into four intervention groups: Tai Chi combined with tDCS (TCT), Tai Chi combined with sham tDCS (TCS), walking combined with tDCS (WAT), and walking combined with sham tDCS (WAS). All participants were assessed at baseline and 12 weeks for global cognitive function, memory, attention, and executive function.

**Results:**

At baseline, there were no significant differences in age, gender, education duration, body mass index, or the Baker Depression Inventory among the four groups (*P* ≥ 0.05). After 12 weeks of intervention, the TCT group showed greater improvements in MOCA scores, memory quotient scores, and digit-symbol coding task reaction time compared to the TCS, WAS, and WAT groups (*P* < 0.05). The TCT group also had a shorter Stroop test color reaction time compared to the WAS and WAT groups (*P* < 0.05), a higher increase in Auditory Verbal Learning Test-immediate recall than the TCS and WAT groups (*P* < 0.05), a shorter visual reaction time than the TCS group (*P* < 0.05), and a shorter sustained attention time compared to the WAT group (*P* < 0.05).

**Conclusion:**

Tai Chi combined with tDCS effectively improves global cognitive performance, memory, execution function, and attention in patients with MCI. These findings suggest the potential clinical use of Tai Chi combined with tDCS as a physical exercise combined with a non-invasive brain stimulation intervention to improve cognitive function in older adults with MCI.

**Clinical trial registration:**

ChiCTR1800015629.

## Introduction

Alzheimer's disease (AD) is an irreversible degenerative disease of the central nervous system. Mild cognitive impairment (MCI) is considered the prodromal stage of AD, mainly manifested as memory or cognitive impairment (1). Epidemiological studies have shown that the prevalence of MCI in people over 65 years is 10–20% worldwide (2). It is estimated that if the onset of AD is delayed by 5 years, the overall prevalence of AD would be reduced by ~50%, thereby significantly reducing the burden on caregivers and institutional care and improving their quality of life (3). The conversion rate of MCI to AD is as high as 32% over 5 years and increases significantly with time (4). Currently, MCI is considered a critical window for the early intervention and rehabilitation of dementia, which can effectively delay the progression to AD (5). Therefore, finding appropriate interventions to improve the cognitive function of MCI and thus reduce the conversion rate of dementia is a major scientific problem that needs to be solved in the field of geriatric health (6).

However, there is a lack of high-quality evidence to support the efficacy of drug interventions in patients with MCI (7). Some drugs have been clinically validated for dementia; however, they have not been shown to improve cognitive function in patients with MCI (8). Thus, the use of non-pharmacological therapies to improve cognitive function in patients with MCI is receiving increasing attention (9).

As MCI causes several intricate neurophysiological abnormalities, comprehensive rehabilitation may be more effective in treating patients with MCI. Recent systematic evaluations support the efficacy of non-invasive brain stimulation (10) and physical exercise (11) on cognitive function in patients with MCI. In 2018, the American Academy of Neurology updated its MCI guidelines to include physical activity as an official recommendation for MCI intervention for the first time (7). A growing number of reviews have highlighted the possibility of combining physical exercise with non-invasive brain stimulation, suggesting that this combination is beneficial for improving cognitive function (12).

A meta-analysis of Tai Chi, a traditional Chinese aerobic exercise, showed a positive effect on improving global cognitive, executive, and memory functions in patients with MCI (13, 14). According to clinical research, 6 months of Tai Chi practice may dramatically enhance test results for memory in older individuals with MCI (15). Moreover, Tai Chi training is helpful for enhancing global cognition, memory, and executive function in older individuals with MCI (16).

Transcranial direct current stimulation (tDCS) is increasingly used as a non-invasive brain stimulation technique to promote functional brain rehabilitation (17). Studies have found that tDCS stimulation of the dorsolateral prefrontal cortex (DLPFC) is effective in improving memory function performance in MCI (18, 19), controlling neurotransmitter release, enhancing synaptic connections, affecting local hemodynamics, and selectively exciting and inhibiting cortical neural activity (20). A randomized controlled trial in which participants received a 4-month tDCS combined with an aerobic exercise intervention showed significant improvements in several cognitive domains compared with both techniques alone (21).

Therefore, we hypothesized that Tai Chi combined with tDCS is beneficial for improving the cognitive function of patients with MCI; however, no targeted studies have confirmed the effect of Tai Chi combined with tDCS on improving the cognitive function of patients with MCI. Therefore, we designed a randomized controlled trial to evaluate the effect of Tai Chi combined with tDCS on cognitive function in patients with MCI.

## Methods

### Design and ethical approval

In this randomized clinical trial, all patients signed an informed consent form before the intervention. The study protocol was reviewed and approved by the Ethics Committee of the Affiliated Rehabilitation Hospital of the Fujian University of Traditional Chinese Medicine (FJTCM) (No. 2017KY-010-02). FJTCM was responsible for the integrity and conduct of this study. The trial was prospectively registered in the Chinese Clinical Trial Registry (ref. ChiCTR1800015629).

### Recruitment and screening

Between April 2018 and February 2020, we recruited participants with MCI from the community in Fuzhou City by posting posters, sending leaflets and brochures, and setting up a recruiting station at the community center. Potential participants first completed a screening questionnaire aimed at assessing their cognitive function to determine their eligibility for the study.

### Sample size

The Montreal Cognitive Assessment (MoCA) score was used as the main indicator of this effect. Referring to the results of previous studies on the effects of Tai Chi and tDCS on cognitive function in patients with MCI, the predicted sample loss rate was ~20%. Using G-Power software with the effect size set at 0.3, power set at 0.8, and α set at 0.05, the calculated required sample size was at least 180 participants.

### Participants

#### Inclusion and exclusion criteria

The inclusion criteria were as follows: participants (1) who met the diagnostic criteria of MCI; (2) with 18 <MoCA score <26; (3) who aged between 50 and 75 years; and (4) did not engage in regular exercise in the last 6 months. The exclusion criteria were as follows: participants (1) with the presence of medical conditions that made exercise unsafe or an inability to exercise (such as patients with uncontrollable blood pressure and hypertension); (2) with a history of severe alcohol and drug abuse; (3) with Baker Depression Inventory (BDI) ≥ 10; (4) with cognitive impairment caused by other reasons; (5) having a history of mental illness (such as personality disorder and schizophrenia), or suffering from severe aphasia, audiovisual impairment, or severe organ failure (such as severe heart failure, respiratory failure, liver failure, and renal failure), history of musculoskeletal system diseases (such as myasthenia gravis, congenital myotonia, fractures, suppurative arthritis, etc.), other motor contraindications (such as unstable angina pectoris, aortic stenosis, etc.), systemic infection, etc.; and (6) who participated in other experiments that influence this study.

#### Randomization and masking

The random grouping sequence of the participants in this study was generated by dedicated statistical staff using Statistical Package for the Social Sciences (SPSS) Statistics 20.0 (IBM, Chicago, IL, USA). Random grouping sequences were stored in a light-impermeable sealed envelope. A total of 180 eligible participants with MCI were randomly assigned into the Tai Chi combined with tDCS group (TCT), Tai Chi combined with sham tDCS group (TCS), walking combined with tDCS (WAT), and walking combined with sham tDCS group (WAS) in order of inclusion. Although blinding is not possible for participants in exercise intervention research, the staff collecting the outcomes and data analysts will have no knowledge of the group assignment. Blinding was performed after the statistical analysis of the study was completed.

## Intervention

All participants with MCI received health education on the etiology and prevention of cognitive impairment prior to the intervention. Health education includes topics, such as MCI and Alzheimer's disease prevention and treatment, as well as the promotion of healthy lifestyles.

### Tai Chi combined with the tDCS group

Participants in the TCT group underwent a 12-week, 24-form Tai Chi training program combined with tDCS. tDCS was performed during the first 20 min of Tai Chi training.

**(1) Tai Chi exercise:** The interventions included three 1-h Tai Chi training sessions per week. Certified instructors delivered Tai Chi training with at least 5 years of experience in directing their respective interventions.

**(2) tDCS stimulation:** The anode of tDCS (The Brain Stimulator v3.0 Deluxe tDCS Kit) was placed over F4 (in EEG 10–20 standard system) with the long axis of the pad pointing toward the vertex of the head. The cathode electrode was positioned over the contralateral eyebrow (Fp1 EEG electrode site, also referred to as the supraorbital position) with the long axis of the pad parallel to the horizontal plane. The electrode assembly consists of two square-shaped electrodes (3 mm × 3 mm). Electrode preparation will involve saturating a sponge electrode with exactly 10 cc of 0.9% saline solution using a marked syringe (5 cc/side) to provide adequate electrode saturation without oversaturation. The same batch of adjustable cross straps is used to secure the sponge pieces and is regularly replaced. Impedances were kept equal to or below 6 kΩ during the whole stimulation session. For the active condition, the current was ramped up to 2.0 mA over 30 s, remained at 2.0 mA for 19 min, and ramped down to 0 mA over 30 s.

### Tai Chi combined with the sham tDCS group

The participants in the TCT group participated in a 12-week 24-form Tai Chi program combined with sham tDCS. Tai Chi training was consistent with those in the TCT group. In the sham tDCS condition, the current was initially ramped up to 2 mA over 30 s and immediately ramped down to 0 mA to prevent the actual stimulation of the target region.

### Walking combined with the tDCS group

The WAT group underwent fitness walking training combined with tDCS for 12 weeks. The exercise intensity was 50–70% of the maximum heart rate for three 1-hour weekly sessions. The tDCS stimulation site, intensity, and duration were consistent with the TCT groups. tDCS was performed during the first 20 min of fitness walking training.

### Walking combined with the sham tDCS group

Participants in the WAS group underwent a 12-week fitness walking training program combined with sham tDCS. Fitness walking training was consistent with the WAT group. In the sham tDCS condition, the current was initially ramped up to 2 mA over 30 s and immediately ramped down to 0 mA to prevent the actual stimulation of the target region.

During the treatment of true and sham tDCS, we have researchers who monitor the entire process, record the subjects' sensations, such as numbness, itching, tingling, and pain felt during treatment at the stimulation site, and report adverse events on time.

### Outcome measures

The outcomes will be assessed at baseline and after 12 weeks of intervention. Examinations were conducted in accordance with a uniform implementation plan and standard operating procedures.

### Primary outcomes

The MoCA and Chinese Wechsler Memory Scale-Revised Memory Quotient (MQ) were used to assess global cognition and memory functions, respectively. The MoCA scores range from 0 to 30, with higher scores indicating better cognitive function. The MQ has a score range of 51 to 150, with higher scores indicating better memory.

### Secondary outcomes

The secondary outcomes included other cognitive subdomain tests performed from baseline to 12 weeks. The episodic memory was assessed using the auditory–verbal learning test (AVLT), while the visual memory function was evaluated using the Rey–Osterrieth complex figure (ROCF). The executive function was assessed using the color-word matching Stroop test, while the attention function was assessed using the Test of Attentional Performance (TAP) (V.2.3, Vera Fimm, Psychologische Testsysteme), which included divided attention (auditory reaction time and visual reaction time), sustained attention, and digit-symbol coding (DSC) tasks.

### Statistical analysis

Data were analyzed using SPSS version 20.0 software (SPSS Inc., Chicago, IL, USA). The categorical data were tested using the chi-square test or Fisher's exact test as appropriate. We performed a Shapiro–Wilk test to check the normality of the continuous variables. For data with a normal distribution, one-way analysis of variance (ANOVA) was used to compare baseline characteristics, and the analysis of covariance (ANCOVA) was used to compare post-intervention changes across the four groups, with age (years) and education (years) as covariates. If there is a statistically significant difference between groups for a certain indicator prior to intervention, this indicator will be included as a covariate in the analysis after intervention. *Post hoc* analysis with Šídák correction was used to explore between-group differences. For non-normally distributed continuous data, we used the Kruskal–Wallis test to compare group differences. Differences were considered statistically significant at a *p*-value of <0.05.

## Results

### Descriptive data

**Figure 1:**
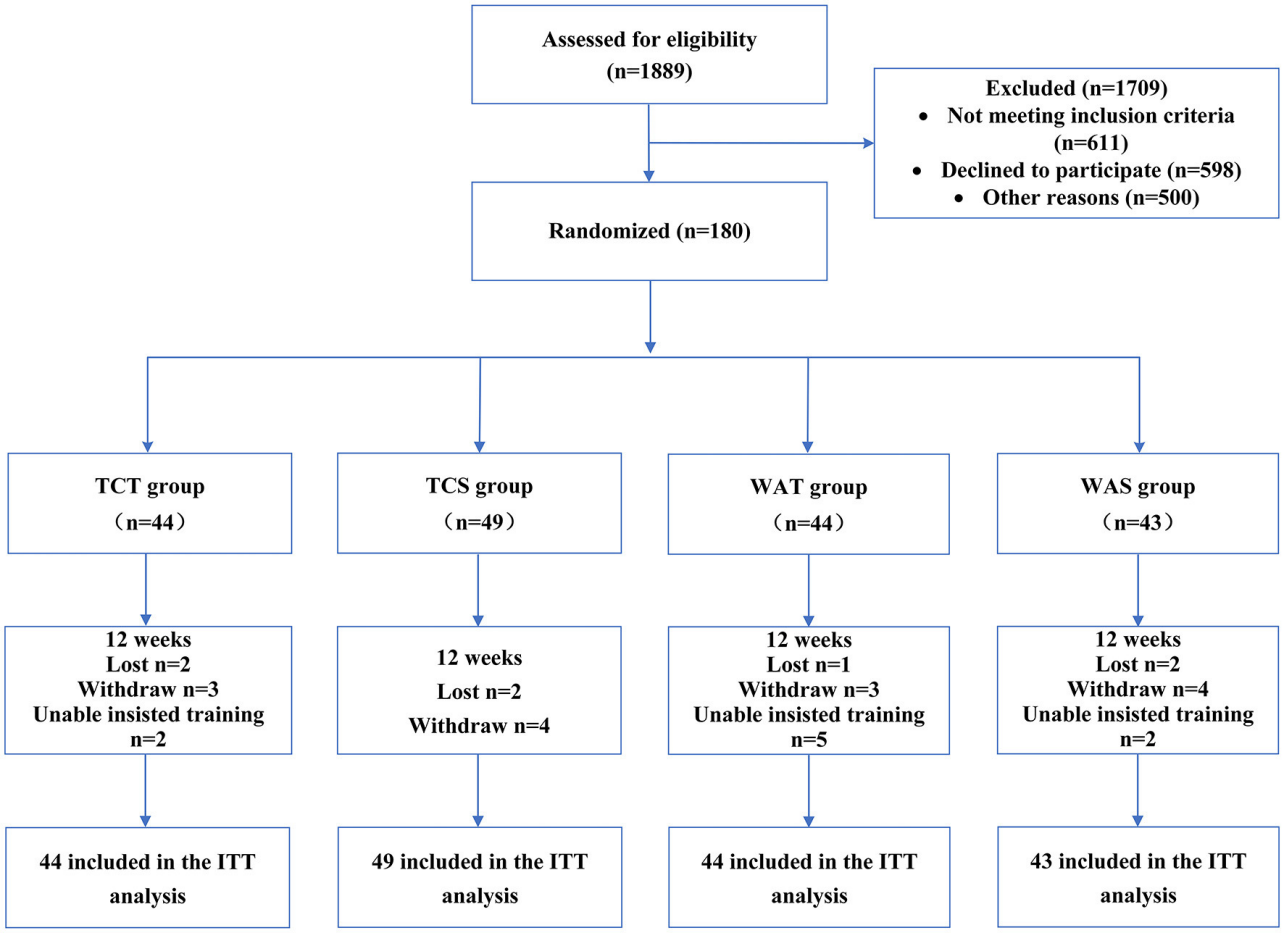
Study CONSORT flow diagram. TCT, Tai Chi combined with tDCS; TCS, Tai Chi combined with sham tDCS; WAT, walking combined with tDCS; WAS, walking combined with sham tDCS; ITT, intention-to-treat.

Overall, 1,889 subjects were recruited for this study from April 2018 to February 2020. Ultimately, a total of 180 eligible subjects were randomly divided into four groups: (i) TCT group; (ii) TCS group; (iii) WAT group; and (iv) WAS group. The flow diagram for this trial is presented in Figure 1.

In total, 30 participants withdrew from the study, of which 7, 6, 9, and 8 withdrew from the TCT, TCS, WAT, and WAS groups, respectively. There was no significant difference in the dropout rate among the four groups (*P* ≥ 0.05). Randomly grouped participants were included in an intention-to-treat (ITT) analysis. The baseline characteristics of the study participants are presented in Table 1.

**Table 1:** Baseline characteristics of participants.

**Characteristics**	**TCT group (*n =* 44)**	**TCS group (*n =* 49)**	**WAT group (*n =* 44)**	**WAS group (*n =* 43)**	**F/Z/χ^2^**	***P*-value**
Age, years	59 [8.75]	61 [8.5]	63 [12.75]	58 [8]	−1.921	0.278^b^
Gender (male/female)	12/32	19/30	21/23	17/26	3.950	0.267^a^
Education duration, years	9.5 [3.75]	9 [5]	9 [4]	9 [4]	−0.598	0.228^b^
BMI, (Kg/m^2^)	22.69 [3.44]	23.78 [3.59]	23.94 [3.47]	24.34 [3.75]	−0.646	0.059^b^
BDI	5.5 [5.75]	5 [5.5]	4 [3.75]	5 [4]	−0.098	0.427^b^

SD, standard deviation; IQR, interquartile range; TCT, Tai Chi combined with tDCS; TCS: Tai Chi combined with sham tDCS; WAT, walking combined with tDCS; WAS, walking combined with sham tDCS; BMI, body mass index; BDI, Baker Depression Inventory.

Data are presented as mean±SD or median [IQR].

^a^Using chi-square test.

^b^Using Kruskal–Wallis test.

### Primary outcomes

**Figure 2:**
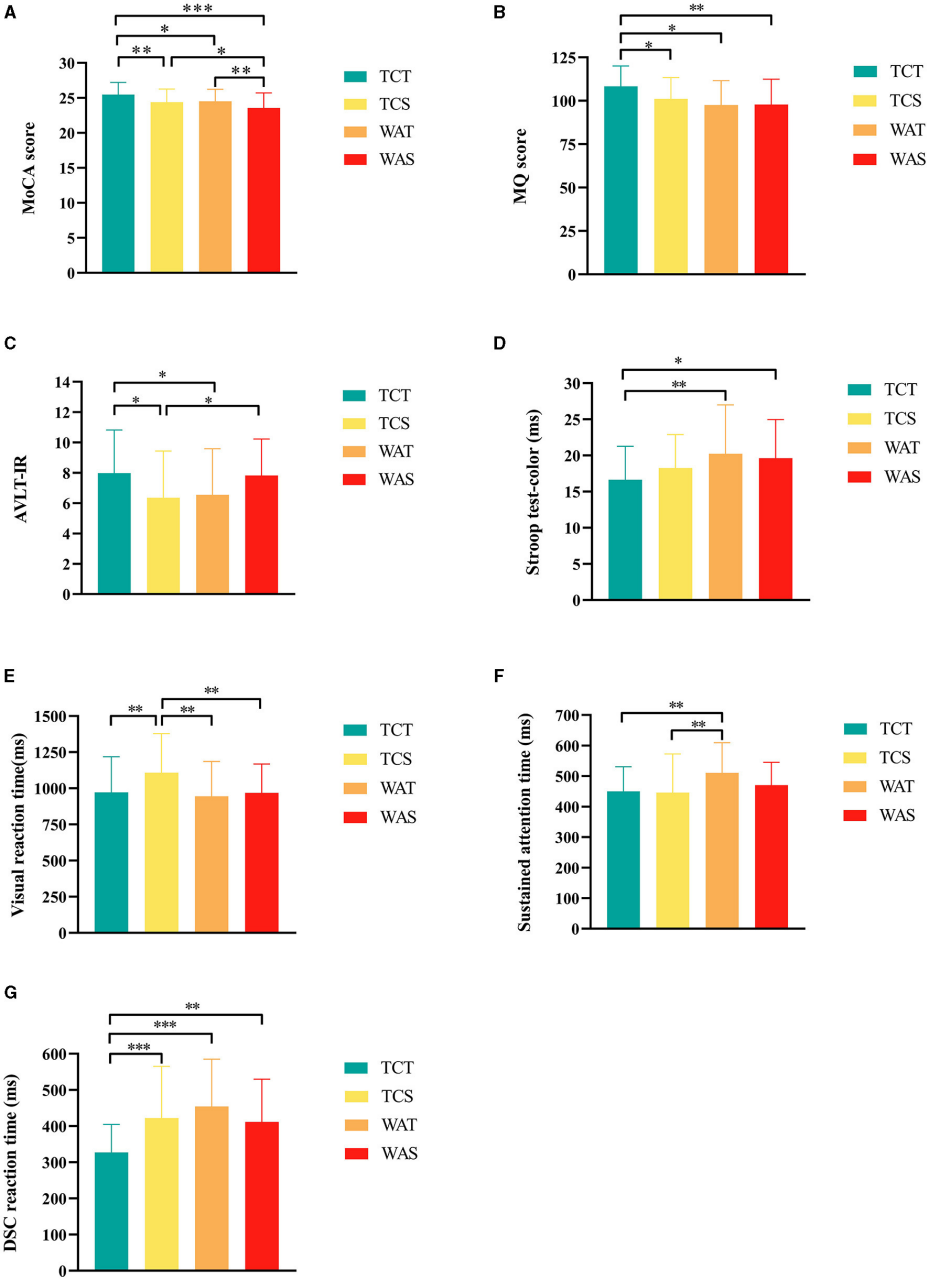
Further *post-hoc* analysis results of outcomes among the groups. **(A)** Montreal Cognitive Assessment (MoCA) score, **(B)** memory quotient (MQ) score, **(C)** auditory verbal learning test-immediate recall (AVLT-IR), **(D)** Stroop test-color, **(E)** Visual reaction time; F, Sustained attention time, **(G)** digit-symbol coding task (DSC) reaction time. *indicate differences of a *P*-value of <0.05, **indicate differences of a *P*-value of <0.01, ***indicate differences of a *P*-value of <0.001. TCT, Tai Chi combined with tDCS; TCS, Tai Chi combined with sham tDCS; WAT, walking combined with tDCS; WAS, walking combined with sham tDCS.

After 12 weeks of intervention, there were statistically significant differences in MoCA scores between groups (*P* < 0.001, F (3,174) = 7.415) (Table 2); furthermore, *post-hoc* analysis showed that TCT was more effective compared with TCS, WAT, and WAS (*P* = 0.012, *P* = 0.032 and *P* < 0.001, respectively) and compared to WAS, the TCS and WAT groups showed higher scores (Figure 2A).

**Table 2:** Comparison of outcomes among groups.

**Characteristics**	**TCT group (*n =* 44)**	**TCS group (*n =* 49)**	**WAT group (*n =* 44)**	**WAS group (*n =* 43)**	**F/Z**	***P*-value**
**Primary outcomes**						
**MoCA**						
Baseline	23.5 [3]	22 [3]	22.5 [3]	22 [3]	−0.095	0.924^b^
12 weeks	25 [2.83]	25 [2]	24.24 [1.61]	23.66 [2.1]	7.415	**<0.001** ^**c**^
**MQ**						
Baseline	97.02 ± 11.84	91.80 ± 15.57	87.41 ± 16.14	91.02 ± 13.07	3.376	**0.020** ^**a**^
12 weeks	108 [17]	100 [16.66]	95.91 [19.01]	99 [18]	3.584	**0.015** ^**c**^
**Secondary outcomes**						
**AVLT-learning score**						
Baseline	19.07 ± 4.88	18.16 ± 5.67	19.48 ± 4.87	19.37 ± 5.03	0.637	0.592^a^
12 weeks	21.90[6.75]	21 [7]	21.80 [3.75]	22.26 [7]	2.557	0.057^b^
**AVLT-IR**						
**Baseline**	6.5 [4]	6 [4]	5 [5]	6 [3]	−1.005	0.315^b^
**12 weeks**	7.24[4.75]	6[5]	5.65[4.25]	7.99[3.16]	3.207	**0.025** ^**c**^
**AVLT-DR**						
Baseline	12 [4]	12 [3]	12 [3]	12 [2]	−0.878	0.380^b^
12 weeks	13[3]	12[3.86]	12.58 [2.73]	12[2.66]	1.130	0.338^c^
**ROCF-copy trial**						
Baseline	32 [4.75]	33 [4]	32 [5.5]	34 [4]	−1.659	0.097^b^
12 weeks	33.5 [4.43]	34 [3.5]	32 [4.93]	34 [3.87]	2.489	0.062^c^
**ROCF-recall trial**						
Baseline	15.48 ± 6.62	14.66 ± 7.78	14.41 ± 5.82	16.19 ± 6.86	0.620	0.603^a^
12 weeks	18[8]	17 [8.09]	15 [10.81]	16.78 [11]	0.571	0.635^c^
**Stroop test color, ms**						
Baseline	17.02 [7.61]	18.94 [8.17]	20.43 [8.54]	19 [7.11]	−0.262	0.793^b^
12 weeks	16.81 [5.51]	18.43 [8.04]	19.17 [5.36]	18.84 [6.9]	2.938	**0.035** ^**c**^
**Stroop test word, ms**						
Baseline	22.64 [8.02]	23.46 [7.92]	26.16 [10.78]	22.79 [6.8]	−0.305	0.760^b^
12 weeks	22.18 [6.36]	21.92 [6.90]	23.87 [10.09]	23.77 [7.74]	2.505	0.061^c^
**Stroop test color-word, ms**						
Baseline	38.59 [10.78]	34.7 [13.68]	36.43 [8.71]	34.16 [11.59]	−0.372	0.710^b^
12 weeks	32.78 [15.35]	33.14 [10.74]	34.13 [12.90]	34.12 [8.73]	0.058	0.982^c^
**Auditory reaction time, ms**						
Baseline	696 [137]	740 [222]	730.22 [216.45]	766 [137]	−0.966	0.334^b^
12 weeks	704 [179.5]	738 [283.69]	808.58 [373.62]	795 [203.51]	2.660	0.050^c^
**Visual reaction time, ms**						
Baseline	940.34 [215.5]	1010 [239.31]	1048.5 [287.25]	1010 [247]	−0.579	0.563^b^
12 weeks	938.29 [180.58]	1076 [327.97]	941.75 [260.57]	965.82 [156]	4.536	**0.004** ^**c**^
**Sustained attention time, ms**						
Baseline	420.96 [74.5]	430 [123.5]	471.22 [101.08]	454 [95]	−0.978	0.328^b^
12 weeks	442.59 [105.34]	447.27 [140.54]	498.5 [102.70]	458.75 [81]	3.609	**0.015** ^**c**^
**DSC reaction time, ms**						
Baseline	330.25 [104.25]	335.5 [161.75]	393.66 [126.37]	360.84 [104]	−1.029	0.303^b^
12 weeks	308.65 [83.04]	415.88 [213.94]	421.64 [153.56]	380 [144.52]	7.846	**<0.001** ^**c**^

SD,standard deviation; IQR, interquartile range; ANOVA, analysis of variance; TCT, Tai Chi combined with tDCS; TCS, Tai Chi combined with sham tDCS; WAT, walking combined with tDCS; WAS: walking combined with sham tDCS; MoCA, Montreal Cognitive Assessment; MQ, memory quotient; AVLT-IR, auditory verbal learning test-immediate recall; AVLT-DR, auditory verbal learning test-delayed recall; ROCF, Rey–Osterrieth complex figure; DSC, digit-symbol coding task.

Data are mean ± SD or median [IQR]. Bold text indicates a *P*-value of <0.05.

^a^Using one-way ANOVA.

^b^Using Kruskal–Wallis.

^c^Using ANCOVA.

We added the pre-intervention MQ scores to the covariates for the analysis. The results showed a statistically significant difference among the four groups (*P* = 0.015, F (3,173) = 3.584) (Table 2), and *post-hoc* analysis showed that TCT was more effective than TCS, WAT, and WAS (*P* = 0.022, *P* = 0.024, and *P* = 0.002, respectively) (Figure 2B).

### Secondary outcomes

In the AVLT test, the scores of AVLT-immediate recall showed statistically significant differences among the four groups (*P* = 0.025, F (3,174) = 3.207) (Table 2), and a *post-hoc* analysis showed that the score of TCT was higher than that of TCS and WAT (*P* = 0.013 and *P* = 0.032, respectively), and the score of WAS higher than TCS (*P* = 0.032) (Figure 2C).

In the Stroop test, the reaction time of the Stroop test color showed statistically significant differences among the four groups (*P* = 0.035, F (3,174) = 2.938) (Table 2), and a *post-hoc* analysis showed that the reaction time of TCT was shorter than that of WAT and WAS (*P* = 0.008 and *P* = 0.022, respectively) (Figure 2D).

In the TAP test, the auditory reaction time showed no statistically significant differences among the four groups (*P* = 0.050, F (3,174) = 2.660) (Table 2), while the visual reaction time showed statistically significant differences among the four groups (*P* = 0.004, F (3,174) = 4.536) (Table 2), and a *post-hoc* analysis showed that the reaction time of TCT was shorter than that of TCS (*P* = 0.007), and the reaction time of TCS was higher than that of WAT and WAS (*P* < 0.001, and *P* = 0.006, respectively) (Figure 2E). The sustained attention time showed statistically significant differences among the four groups (*P* = 0.015, F (3,174) = 3.609) (Table 2), and a *post-hoc* analysis showed that the reaction time of WAT was higher than that of TCT and TCS (*P* = 0.007, *P* = 0.003, respectively) (Figure 2F). The DSC reaction time showed statistically significant differences among the four groups (*P* < 0.001, F (3,174) = 7.846) (Table 2), and a *post-hoc* analysis showed that the reaction time of TCT was shorter than that of TCS, WAT, and WAS (*P* < 0.001, *P* < 0.001, and *P* = 0.004, respectively) (Figure 2G).

In the ROCF-copy trial test, no statistically significant differences were seen between the four groups (*P* = 0.062, F (3,174) = 2.489). In the ROCF-recall trial test, no significant differences were seen between the four groups (*P* = 0.635, F(3,174) = 0.571).

## Discussion

To the best of our knowledge, this is the first study to focus on the effects of Tai Chi exercise combined with tDCS on cognitive function in patients with MCI. Among patients with MCI, Tai Chi combined with tDCS was more effective than the other three groups in terms of MoCA, MQ scores, and DSC after 12 weeks of intervention. The results of this study suggest that Tai Chi combined with tDCS improves global cognitive function, memory, and attention in patients with MCI.

Currently, most published tDCS studies on patients have focused on the combination of tDCS technology and cognitive training (22). Only one small-sample study has investigated a combination of tDCS and Tai Chi exercises (23). This study observed the comprehensive effects of Tai Chi combined with tDCS on the dual-task gait performance and cognitive function in patients with MCI and found that 12 weeks of Tai Chi combined with tDCS were superior to Tai Chi combined with sham tDCS in improving the dual-task gait. However, the cognitive function results were not significant after correction. In contrast, our study showed a significant improvement in the cognitive function of patients with MCI after 12 weeks of Tai Chi combined with tDCS. The reason for this difference may be the relatively small sample size (20 cases) of the previous study, which may not have demonstrated the effectiveness of Tai Chi combined with tDCS technology in improving cognitive function.

The results of our study showed that, in terms of global cognitive function, the TCT was more effective than the TCS, WAT, and WAS. There was no significant difference between TCS and WAT, although TCS and WAT showed a more significant improvement than WAS. As an aerobic exercise, Tai Chi induces functional neuroplastic changes in the brain, which prepares tDCS to target and regulate local brain areas (20). Improvement in cognitive function with Tai Chi is a gradual process. Therefore, it is believed that combining the two may have stronger effects on cognitive improvement by stimulating local neural networks in the brain in a more targeted and focused manner and by promoting brain neuroplasticity.

Furthermore, when compared to walking, Tai Chi calls for subjects to pay more attention to the exercise process, the purpose of following their bodies, and the ability to notice tiny changes in their bodies and minds as they perform complicated moves. This may be one of the reasons why Tai Chi combined with tDCS is more beneficial for improving cognitive function in patients with MCI. Therefore, we believe that Tai Chi combined with tDCS may be more effective in improving global cognitive function.

Regarding the memory function assessment, the MQ scores of the Tai Chi combined with the tDCS group were significantly higher than those of the other three groups. This suggests that Tai Chi combined with tDCS has a positive effect on memory function in patients with MCI, which is consistent with the findings of previous studies. Ward et al. (21) found that cognitive training combined with non-invasive brain stimulation significantly improved participants' learning and working memory scores compared to cognitive training alone in a study of 318 healthy adults over 48 sessions over 16 weeks, suggesting that the combined intervention could improve learning and memory function in adults.

Although the present study and Ward's (21) study differed in the population of the intervention and modality of the exercise, both found that the multimodal intervention significantly improved the memory function of the participants compared with the single intervention modality. This finding suggests that there may be a synergistic effect of the combined intervention program that facilitates the improvement of memory function in patients with MCI and may provide a reference for future studies to improve memory function in patients with MCI.

Attention is the basis of memory production and is closely related to other cognitive domains (24). However, the process of attentional impairment in patients with MCI is subtle and is typically accompanied by decreases in other cognitive domains, including memory function (25). Several studies have shown that aerobic exercise improves attention in patients with mild cognitive impairment. According to a meta-analysis of 632 participants, Tai Chi can help normal older persons improve cognitive function in several areas, including attention (26). In another randomized controlled study on the effects of tDCS on attention, 26 subjects received tDCS/sham tDCS stimuli in the right posterior parietal cortex and underwent attentional network tests before and after the intervention, showing that tDCS significantly improved the subjects' orienting performance of attentional functions compared with pseudo-stimuli, whereas executive control effects were not affected (27). In this study, the subjects' attention was assessed using TAP 2.3, an attention test system. The results of this study showed that the Tai Chi combined with the tDCS group had significantly higher sustained attention scores than the other three groups, indicating that Tai Chi combined with tDCS is more beneficial for improving the attention of patients with MCI.

An increasing body of research indicates that tDCS and aerobic exercise have synergistic effects, whereby aerobic exercise causes neuroplastic changes across the brain, intensifies local targeting of tDCS to the brain, and improves cognition (20, 28). Aerobic exercise encourages the production of neurotrophic factors, boosts neurogenesis and synaptogenesis, and delays cognitive impairment. Non-invasive brain stimulation integrates regenerated neural regions into functional brain circuits. Consequently, multimodal therapies emphasizing functional compensatory mechanisms can help enhance cognitive performance in individuals with cognitive decline and promote neurological compensation. Studies have shown that the combination of tDCS and aerobic exercise has beneficial effects (29, 30). Anodal tDCS stimulation of the prefrontal cortex combined with aerobic exercise improves cognitive function (24). In this study, Tai Chi as a moderate-intensity aerobic exercise combined with tDCS had a more positive effect in improving the global cognitive function, memory function, and attention compared to TCS, WAT, and WAS, and the results support the above view.

This study used a strict randomized controlled approach and strengthened quality control during the intervention to minimize possible bias with the aim of providing reliable clinical evidence. However, this study has some limitations. First, the implementation site of this study was mainly outdoors, making it impossible to avoid the effect of rain on Tai Chi and walking training. Although subjects were asked to practice at home on their own, the efficiency of the intervention was inevitably affected. Second, no follow-up study was conducted, and whether there is a long-term effect of Tai Chi combined with tDCS remains to be explored. Future large-scale, well-conducted, randomized controlled trials with appropriate comparison groups and follow-up arrangements are required to evaluate the long-lasting effects of Tai Chi combined with tDCS on cognitive health.

## Conclusion

This study found that Tai Chi combined with tDCS was more beneficial in improving the global cognitive function, memory function, execution function, and attention in patients with MCI. These non-pharmacological interventions are easy to implement, time- and cost-effective, safe, and have no serious side effects. Therefore, we believe that Tai Chi combined with tDCS is an effective intervention for patients with MCI.

## Data availability statement

The raw data supporting the conclusions of this article will be made available by the authors, without undue reservation.

## Ethics statement

The studies involving human participants were reviewed and approved by Ethics Committee of Rehabilitation Hospital Affiliated to Fujian University of Chinese Medicine. The patients/participants provided their written informed consent to participate in this study.

## Author contributions

WL, JT, and JL contributed to the conception, the design of the study, and revised the manuscript. JL, ZL, and JH were responsible for coordinating and monitoring the process. YX and JZ performed the statistical analysis and wrote the first draft of the manuscript. HL, ZQ, and MW managed the recruitment and data analysis. All authors contributed to the manuscript revision, read, and approved the submitted version.
